# Effects of anabolic-androgens on brain reward function

**DOI:** 10.3389/fnins.2015.00295

**Published:** 2015-08-26

**Authors:** Emanuela Mhillaj, Maria G. Morgese, Paolo Tucci, Maria Bove, Stefania Schiavone, Luigia Trabace

**Affiliations:** ^1^Department of Physiology and Pharmacology, Sapienza University of RomeRome, Italy; ^2^Department of Clinical and Experimental Medicine, University of FoggiaFoggia, Italy

**Keywords:** anabolic androgenic steroid, reward, dopamine, serotonin, psychosis spectrum disorders, depression

## Abstract

Androgens are mainly prescribed to treat several diseases caused by testosterone deficiency. However, athletes try to promote muscle growth by manipulating testosterone levels or assuming androgen anabolic steroids (AAS). These substances were originally synthesized to obtain anabolic effects greater than testosterone. Although AAS are rarely prescribed compared to testosterone, their off-label utilization is very wide. Furthermore, combinations of different steroids and doses generally higher than those used in therapy are common. Symptoms of the chronic use of supra-therapeutic doses of AAS include anxiety, depression, aggression, paranoia, distractibility, confusion, amnesia. Interestingly, some studies have shown that AAS elicited electroencephalographic changes similar to those observed with amphetamine abuse. The frequency of side effects is higher among AAS abusers, with psychiatric complications such as labile mood, lack of impulse control and high violence. On the other hand, AAS addiction studies are complex because data collection is very difficult due to the subjects' reticence and can be biased by many variables, including physical exercise, that alter the reward system. Moreover, it has been reported that AAS may imbalance neurotransmitter systems involved in the reward process, leading to increased sensitivity toward opioid narcotics and central stimulants. The goal of this article is to review the literature on steroid abuse and changes to the reward system in preclinical and clinical studies.

## Introduction

Anabolic-androgenic steroids (AAS) are synthetic compounds derived from testosterone, which is the main male hormone. The binding of testosterone to androgen receptors has anabolic and androgenic effects. During puberty, the increase in testosterone levels contributes to linear growth augmentation, as well as muscle mass accumulation (Bhasin et al., 1996, 2001; Brower, 2002; Kuhn, 2002) by inducing hypertrophy without changes in the absolute number of both Type 1 and 2 muscle fibers (Sinha-Hikim et al., 2002). Testosterone also acts by increasing the number of muscle progenitor cells (Sinha-Hikim et al., 2003) and promoting their myogenic differentiation (Singh et al., 2003, 2006). Testosterone promotes mitochondrial biogenesis, improves net oxygen delivery to the tissue by increasing red cell mass and tissue capillarity, and facilitates oxygen unloading from oxyhemoglobin (Coviello et al., 2008; Gupta et al., 2008). The idea of designing and developing steroids with anabolic properties arose during the 1930s soon after the identification and isolation of the hormone androsterone by the German investigator Butenandt, who collected this compound from thousands of liters of pooled human urine derived from a number of military service volunteers. Most of the AAS used before the 1990s were pharmacological agents approved for medicinal or veterinary use. By the 1990s, various androgen precursors became nutritional supplements. Androgen precursors are either inactive or weak androgens that the body converts into potent androgens. These include naturally occurring precursors to testosterone, such as 4-androstenediol, 5-androstenediol, 4-androstenedione, and dehydroepiandrosterone, as well as precursors to synthetic AAS including 4-norandrostenedione, 4-norandrostenediol, and 5-norandrostenediol, which the body converts to nandrolone (Pope et al., 2014). Other synthetic AAS, such as 17-desmethylstanozolol, methylclostebol, and methyltrienolone have been recently introduced into the market as dietary supplements. These “designed” steroids have not undergone toxicological or safety testing in humans or animals. Thus, they potentially represent an even more serious health risk than the more traditionally used AAS.

## Medical use

From a clinical standpoint, AAS are commonly prescribed to treat several disorders, such as the androgen deficiency syndromes (Conway et al., 2000), hereditary angioedema, hematological disorders (Shahidi, 2001), catabolic conditions, such as some types of cancer-related cachexia (Langer et al., 2001), metabolic dysfunctions induced by severe burn (Hart et al., 2001), inflammatory pulmonary diseases (Ferreira et al., 2001), radiation therapy, and AIDS-associated malnutrition (Basaria et al., 2001; Polsky et al., 2001). Less common medical uses of AAS deal with heart and renal failure (Basaria et al., 2001). Contrasting data exists in the literature regarding the use of AAS in the treatment of androgen deficiency in aging males, infertility, sexual dysfunctions or impotence, as well as post-menopausal syndrome in women. Thus, while a review of Morley (2001) points toward therapeutic effects on *libido* and menopause-induced sarcopenia, Conway et al. (2000) consider their therapeutic application in these pathological conditions as ≪misuse of androgens≫. Hence, according to the state of the art presented in their review, they reported no indication for androgen therapy in male infertility because of its suppressing effect on spermatogenesis. Importantly, there is no evidence in available literature that AAS abuse or dependence might develop from the legitimate medical use of AAS.

## Non-medical use

The use of AAS for non-medical intentions can easily determine abuse and lead to dependence. When used by athletes, AAS can improve performance to levels obtainable by virtually any other combination of non-chemical solutions provided by modern sport techniques (Noakes, 2004). Generally, supra-pharmacological doses of AAS act either by a direct mechanism, promoting an increase in mass, force, speed of muscular contraction, and recovery after intense physical exercise (Tremblay et al., 2004) or by an indirect pathway through erythropoietic stimulation, leading to increased synthesis of 2,3-diphospholglycerate and tissutal oxygen transfer facilitation (Shahidi, 2001). Consumption of high doses of AAS typically consists in 6–12 week cycles, followed by a 6–12 week period of wash-out. These patterns of AAS use may easily precipitate in periods of continuous consumption without any AAS-free intervals due to the fact that abusers try to assure their muscle gains while avoiding withdrawal symptoms (Brower, 2002; Kuhn, 2002). Several other drugs are frequently associated with the use of supra-pharmacological doses of AAS by abusers that are designed to increase their effects, diminish side effects or avoid detection by urine testing (Wichstrom and Pedersen, 2001). The abuse of other illicit drugs, such as amphetamines and opioids, has also been shown to be strengthened by AAS use (Arvary and Pope, 2000). Moreover, such abuse might reinforce the occurrence of adverse substance interactions. In particular, in the case of AAS and amphetamine association, the overdose potential appears to be increased, due to cardiotoxicity (Thiblin et al., 2000). The contemporary consumption of AAS and bromocriptine, used to rapidly reduce body fat and total weight, has been described as the cause of a syndrome characterized by syncopal episodes and atrial fibrillation (Manoharan et al., 2002).

Populations of adolescents and young adults have been the subject of several clinical studies that explore the prevalence of AAS misuse and abuse. Irving et al. (2002) conducted a study on a population of 4746 middle and high school students from public schools of Minneapolis completing surveys and anthropometric measurements as part of a population-based study of eating patterns and weight concerns among teenagers (Project EAT: Eating Among Teens). They observed that steroid use was more common in non-Caucasian males and in middle school students as compared to high school. In males, steroid use was associated with poor self-esteem, higher rates of depressed mood and attempted suicide, poor knowledge and attitudes about health, greater participation in sports emphasizing weight and shape, greater parental concern about weight, and higher rates of eating disorders and substance abuse. In a study by Wichstrom and Pedersen (2001), a representative sample of 8877 Norwegian youths (15–22 years of age) was surveyed. Results showed that AAS use did not vary according to sport involvement or demographics. Moreover, AAS use was associated mainly with the abuse of marijuana, aggressive-type conduct problems and eating disorders.

## Adverse effects

The severity and impact of side effects induced by AAS abuse depend on a wide range of factors, such as dose, duration of administration, possible consumption of a combination of AAS, as well as gender and age of the abusers. Data on the impact of sustained administration, failed to show any documented adverse events associated to a single episode of acute consumption of supra-pharmacological doses of AAS. Their abuse has been shown to be associated to greater effects on physical performance in younger individuals and women, together with increased incidence and risk of developing serious side events (Kindlundh et al., 1999). Few data exist on the risk of side effects linked to long-term use of high-dose of AAS for non-therapeutic purposes (Parssinen and Seppala, 2002). Cardiovascular complications have been widely described in AAS abusers, including the occurrence of arrhythmic events (Furlanello et al., 2003). In a recent *post-mortem* study that compared 87 deceased men positive for AAS with 173 control subjects (Far et al., 2012), AAS users showed significantly greater cardiac mass.

In another clinical investigation, ventricular hypertrophy, associated with fibrosis and myocytolysis, was detected after cardiac death in four AAS users (Montisci et al., 2012). Also, controlled studies realized by echocardiography (Krieg et al., 2007; Hassan et al., 2009; Baggish et al., 2010) or by cardiac magnetic resonance imaging (Luijkx et al., 2013) have demonstrated lower ventricular ejection fractions and reduced diastolic tissue velocities in AAS users.

Pathological effects on urogenital and reproductive systems have been reported. In particular, hypogonadotropic hypogonadism with consequent testicular atrophy in men and development of inhibitory mechanisms for FSH and LH production in women have been described in selected populations of AAS abusers (Anderson and Wu, 1996; Dohle et al., 2003). Increased virility and lowering of voice tone, irregular menstruation with infertility, decreased breast size, hypertrophic clitoris, and increased sexual desire have also been described in a population of female AAS abusers (Franke and Berendonk, 1997; Kutscher et al., 2002). Other complications include liver damage and hepatitis (Tanaka et al., 2000), insulin-resistance secondary to glucose intolerance with alterations of thyroid function (Yesalis et al., 2000), increased risk of infectious diseases caused by inappropriate use of syringes and non-protected sexual relations among AAS users (Aitken et al., 2002). Although several studies point toward a reversibility of undesirable AAS-induced effects following suspension, they can become irreversible complications with prolonged AAS abuse (Kutscher et al., 2002).

## Psychiatric effects

AAS are universally recognized to have psychoactive effects (Yates, 2000). Although some spared studies have reported their therapeutic use in depression to improve mood and anergia (Rabkin et al., 2000), most evidence points toward the association of AAS with depression, mania, psychosis, suicide and increased aggression leading to violence and, in extreme cases, to homicide (Pope and Brower, 2000; Pope et al., 2000; Thiblin et al., 2000). Indeed, suicide and homicide have been shown to be the main cause of premature deaths among steroid users and, in particular, in the teen population (Thiblin et al., 1999b). Although this does not imply that all steroid users will suffer crippling depression or homicidal rage, steroids appear to strongly contribute to psychiatric dysfunctions in susceptible individuals.

Globally, the prevalence of AAS-induced psychiatric disorders has been hard to evaluate and determine, because of sampling biases in clinical case reports. In a review of Pope et al., (Pope et al., 2000), summarizing four prospective, placebo-controlled trials, it has been reported that at least 5% of AAS users will experience AAS dose-dependent maniac or hypomaniac episodes (Pope and Brower, 2000). However, this estimated percentage appears to be influenced by the fact that in most controlled trials, it is not possible to completely mimic the extreme doses and combinations of AAS taken by abusers for ethical reasons. Thus, estimated rates of AAS-induced psychiatric alterations are probably even higher. This is also due to the fact that other factors can increase the likelihood of psychiatric consequences of AAS abuse, such as the presence of a positive psychiatric anamnesis, alcohol, or other drug use (Dean, 2000) as well as other medical comorbidities. For example, in a case-report of Morton et al. (2000), the authors described the case of a man suffering from Axis II psychopathology, who developed severe psychosis after receiving therapeutic doses of an anabolic steroid for burn injuries in combination with lorazepam and opioids.

Psychological motivations contributing to anabolic steroid use and abuse have received little attention in psychiatric literature. Clinical studies demonstrate that steroids are used in part to deal with an earlier trauma, such as childhood physical or sexual abuse (Porcerelli and Sandler, 1995).

## Effects on the brain reward function: dependence and addiction potential

**Figure 1:**
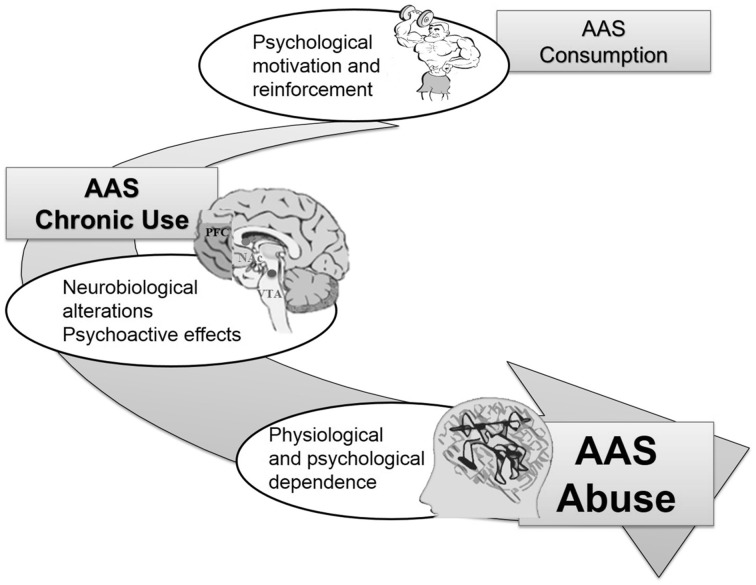
**Main clinical observations linking AAS consumption to AAS addiction**.

The data in the literature show no documented cases of dependence induced by AAS use at therapeutic doses. This suggests that dependence is likely associated to the use of higher doses of AAS (Long et al., 2000; Thiblin et al., 2000; Haupt, 2001; Brower, 2002; Kutscher et al., 2002); Figure 1 graphically represents this hypothesis. However, molecular mechanisms leading to AAS-induced dependence are still unclear.

In a review of the scientific literature published between 1988 and 1998 (Brower, 2000), AAS dependence was defined as a diagnosable mental disorder. Between 1999 and 2000, two more diagnostic studies of AAS dependence were published (Midgley et al., 1999; Brower, 2000).

A “withdrawal syndrome” induced by AAS abuse has been clearly described, consisting mainly of depressed mood, fatigue, AAS craving, restlessness, anorexia, insomnia, and decreased *libido* lasting for several weeks or months (Brower, 1997, 2000). In the 1980s, Tennant et al. (1988) described a case report on which a model of a biphasic course of withdrawal was proposed. The initial phase of the AAS-induced withdrawal (lasting for about 1 week) seemed to be comparable to opioid-induced withdrawal, while the second phase was mostly characterized by clear depressive symptoms and craving (Tennant et al., 1988).

Considerable evidence suggests that AAS dependence might share crucial mechanisms of opioid dependence in humans. In 1989, Kashkin and Kleber (1989) posited that AAS dependence might partly arise via an opioidergic mechanism, through which AAS might enhance the activity of central endogenous opioids, and AAS withdrawal would lead to a decrease in this activity and a subsequent acute hyperadrenergic syndrome (Kashkin and Kleber, 1989). This posited link between AAS and opioids was later confirmed by a large number of observations indicating that AAS users seem to be particularly at risk for developing opioid abuse or dependence (McBride et al., 1996; Wines et al., 1999). Additional clinical studies provided evidence that AAS might decrease the analgesic action of both metamizol and morphine (Philipova et al., 2003).

In 2009, a study by Kanayama et al. (2009) added further evidence for a relationship between AAS and opioids. In the population included in that study, opioid abuse or dependence began either before or after the onset of AAS use, suggesting the possibility that these forms of substance abuse might arise from a common molecular pathway (Kanayama et al., 2009). However, in a study of Negus et al. (2001), authors could not detect any withdrawal phenomena following administration of high doses of AAS (Negus et al., 2001).

AAS seem to act through a more modest reinforcement mechanism compared to cocaine or heroin and resembles the reinforcement mechanism described for caffeine, nicotine, and benzodiazepines. In 2002, Brower (2002) proposed a 2-stage model of steroid dependence. In Stage 1, anabolic effects of AAS provide the initial input and motivation for AAS consumption. Stage 2 deals with consequent chronic use, following which physiological and psychological dependence may develop, thereby making it increasingly difficult for users to quit. Psychoactive effects, such as mood changes and increases in aggressive behavior, characterize this stage of dependence. Diagnostic and Statistical Manual of Mental Disorders criteria for AAS dependence are met and users are not able to stop or discontinue AAS consumption. In Stage 2, addiction treatment may be required, especially when AAS abuse is associated with other substance dependence, such as alcohol, opioids, or amphetamine abuse (Brower, 2002). Arvary and Pope (2000) investigated this phenomenon in a clinical study, including 227 patients admitted to a private facility for dependence on heroin or other opioids. Results of this study strongly suggested that these patients were introduced to opioids through AAS use and bodybuilding physical activity. In particular, 81% of them first purchased opioids from the same drug dealer who had sold them AAS; 67% were introduced to opioids by a fellow body-builder; 86% first used opioids to reduce insomnia and irritability induced by AAS, and 67% used opioids to diminish depression feelings induced by withdrawal from AAS (Arvary and Pope, 2000).

A second model, explaining mechanisms leading to AAS dependence, has also been proposed (Bahrke and Yesalis, 1994). This model holds that AAS-dependence development occurs specifically in socio-cultural contexts that are likely to motivate certain individuals, particularly men, to attain large and strong muscles by frequent and intensive training sessions. These training sessions also improve mood and self-esteem and are generally associated with very strict and controlled dietary regimens. Thus, AAS-induced muscle-active effects might underlie the reinforcing actions of these compounds (Midgley et al., 1999) and the compulsive features of AAS use seem to strengthen the likely compulsive patterns of training and diet. Studies to elucidate mechanisms leading to AAS dependence have also included surveys of current and former AAS users, recruited from gyms, websites, and physicians. Brower et al. (1990) reported numerous criteria for psychoactive substance dependence in a survey of eight AAS abusers, including continued use despite adverse side effects, and withdrawal symptoms (Brower et al., 1990).

Specific dysfunctions of the various components of the brain reward system have been described in clinical studies. For example, alterations in levels of monoamine metabolites, neurohormones, and neuropeptides, which play a crucial role in the reward mechanism, have been investigated in the cerebrospinal fluid of subjects who received methyltestosterone (MT) with respect to placebo-treatment (Daly et al., 2001). Results showed that levels of 5-hydroxyindolacetic acid (5-HIAA) increased while 3-metoxy-4-hydroxyphenylglycol (MHPG) levels decreased in cerebrospinal fluid, following MT administration. In particular, changes in cerebrospinal fluid 5-HIAA significantly correlated with the activation of specific psychiatric symptom cluster scores. In addition, according to this study, a decrease in cerebrospinal fluid MHPG may derive from reduced norepinephrine clearance, even though authors did not detect any significant correlations between changes in MHPG levels and the development of clear psychiatric symptoms, suggesting a less crucial role for noradrenergic changes in this process. An increase in substance P levels and vasopressin (Hallberg et al., 2000; Harrison et al., 2000), as well as dysfunctions of the central opioid system (Schlussman et al., 2000), have been proposed as playing a potential role in the development of aggressive behavior after AAS abuse.

Multiple factors have been associated with the induction of dependence in AAS users, such as low endogenous levels of testosterone. Indeed, it has been demonstrated that women, adolescents and elderly subjects have a lower probability of developing AAS dependence (Wood et al., 2004). Among possible risk factors for dependence development, the most relevant appears to be participation in competitive sports with intense and repetitive physical exercise (Kanayama et al., 2003b). Some investigators have also suggested that personality psychopathology may be a risk factor for AAS abuse. Yates et al. (1990) reported that AAS users and weight lifters had a higher prevalence of histrionic, antisocial, and borderline personality traits than community controls. Although a growing number of reports, current knowledge of molecular mechanisms leading to AAS dependence in humans remains limited. In this regard, the reinforcing effects of AAS may also be biased by intensive physical exercise and by increased narcissistic self-esteem arising from the fulfillment of the desired body appearance. On the other hand, many users practice “stacking” consumption, consisting in the contemporary mixed use of multiple steroids.

Since it has been reported that around 96% of users combine AAS with other drugs in order to relieve non-medical steroid side effects (Parkinson and Evans, 2006), pharmacodynamics, and pharmacokinetic interaction studies are surely warranted, although hardly feasible, in order to exclude further bias.

## Behavioral and neurochemical responses to AAS administration in animal models

Preclinical studies have contributed in evaluating the impact of AAS exposure on neurochemical mechanisms underlying AAS-induced behavioral outcomes. Animal studies offer a direct measure of behavioral parameters under conditions where age and sex of the subjects, along with AAS administration, are established by the investigator. In this section, we will focus our attention on the data in the literature from animal models employing different AAS exposure paradigms, frequently used to model human abuse patterns. In particular, we will review laboratory animal research findings to assess AAS-induced behavioral effects, such as aggression and reward. Moreover, we will highlight studies that have reported neuronal pathways and signaling molecules involved in these behaviors.

## Aggression

Behavioral human studies linking AAS abuse and aggression have confounding factors, such as regimen (multiple steroids over a cycle of use), co-administration with other drugs of abuse and inaccurate measures of behavior simulated by subjective reports (McGinnis, 2004). Conversely, experimental designs in animals that correlate AAS exposure and aggression are less equivocal.

The resident-intruder test is a common paradigm for assessing aggression. Initial studies on animal models have reported that long-term exposure to high doses of testosterone raised levels of aggression in gonadally intact rats and re-established aggression in castrated rats (Lumia et al., 1994). However, indices of aggressive responses depend on environmental context, social cues, sex and hormonal status of the intruder, age of exposure, physical provocation, and type of AAS administered (Clark and Henderson, 2003; Lumia and McGinnis, 2010). Hence, studies in rats showed that AAS-treated males demonstrated a different predisposition for aggression when tested in three different environments (home cage, opponent cage, or neutral cage) (Christie and Barfield, 1979; Lumia et al., 1994; Breuer et al., 2001; Farrell and McGinnis, 2003). Adult male rats receiving high doses of AAS are more aggressive toward the intruder in their home cage and displayed lower levels of aggression in either opponents or neutral cages (Breuer et al., 2001; Farrell and McGinnis, 2003). Investigators extended their interest to other experimental factors demonstrating that AAS-treated rats are typically more aggressive toward intact rather than castrated rats, as well as toward ovariectomized rather than sexually receptive females (Breuer et al., 2001; Farrell and McGinnis, 2003; Cunningham and McGinnis, 2006, 2007). McGinnis et al. (2002a), showed that 12 weeks of testosterone propionate exposure enhanced inter-male aggression in adult rats after physical provocation in the form of a mild tail pinch. Moreover, the environmental and social discriminating cues described above failed to alter testosterone-induced aggressive responses to physical provocation (McGinnis et al., 2002a,b). While testosterone clearly increases aggression, conflicting results have been reported in the literature concerning other commonly abused AAS (stanozolol, nandrolone decanoate, boldenone undecylenate) tested either in combination or individually. Salas-Ramirez et al. (2010) tested whether a 2-week administration of an AAS cocktail containing testosterone cypionate, nandrolone decanoate, and boldenone undecylenate had dissimilar behavioral consequences when drug exposure occurred during adolescence or adulthood. Higher aggression levels were observed in male Syrian hamsters exposed to an AAS cocktail compared to controls, regardless of age treatment (Salas-Ramirez et al., 2010). On the other hand, stanozolol failed to induce aggressive behavior in gonadectomized and intact rats and mice (Clark and Barber, 1994; Martinez-Sanchis et al., 1996; McGinnis et al., 2002a). More conflicting results have been reported by using nandrolone decanoate. Long et al. (1996) showed increased levels of aggression in Sprague–Dawley rats receiving chronic nandrolone decanoate, while no effect has been evidenced in Wistar rats (Zotti et al., 2014). Accordingly, adult rats exposed to mild physical provocation demonstrated decreased inter-male aggression when treated with stanozolol, while no effects of nandrolone have been reported (Breuer et al., 2001; Farrell and McGinnis, 2003). Regardless of the experimental methodologies employed to assess aggression, these findings suggest that strain, AAS chemical composition and regimen reflect the diversity of supra-therapeutic AAS exposure on behavioral responses in animals.

Several studies in preclinical models of aggression have investigated the AAS effects on the neurochemical changes in specific brain areas related to this behavior.

High aggression is often associated to decreased serotonin (5-HT) neurotransmission. Although this may account for high aggression as an individual feature, it has been suggested that serotonergic activity is probably higher during performance of aggressive behavior (van der Vegt et al., 2003).

In particular, testosterone propionate exposure decreased both 5-HT and 5-HT metabolite, 5-HIAA, in the hippocampus but not in the striatum or in the frontal cortex of adult rats (Bonson et al., 1994). Moreover, the aggressive behavior of dominant rats was decreased by treatment with selective agonists of 5-HT_1A_, 5-HT_1B_, and 5-HT_2A∕2C_ receptors (Bonson et al., 1994). A significant decrease in 5-HT_1A_ and 5-HT_1B_ receptors immunoreactive staining has been shown in the latero-anterior hypothalamus and amygdala of hamsters treated with a mixture of AAS (Grimes and Melloni, 2005; Ricci et al., 2007). However, no decrease in the number of 5-HT_1A_ receptor-expressing neurons and an increase in 5-HT_2A_ receptor immunoreactivity have been reported in the hypothalamus (Ricci et al., 2006; Schwartzer et al., 2009). Ambar and Chiavegatto (2009) have reported reduced 5-HT_1B_ mRNA levels in the hippocampus, hypothalamus, amygdala, and prefrontal cortex of nandrolone-treated mice suggesting that the serotonergic tone in these brain areas has a pivotal role for AAS-induced aggression in rodents (Ambar and Chiavegatto, 2009).

## Reward

The data in literature highlight the potential for AAS addiction in humans (Kashkin and Kleber, 1989; Brower et al., 1990, 1991; Brower, 2002; Wood, 2004). Nevertheless, it is difficult to separate the direct rewarding effects of AAS from the psychological dependence of users on their physical appearance, muscular strength, and athletic performance. Hence, studies in animal models are a useful tool when examining androgen-reinforcing properties in conditions where anabolic effects and athletic performance are not relevant. Conditioned place preference (CPP) and self-administration are relevant experimental paradigms used to study reward in an experimental condition (Wood, 2004; Koob, 2006). Several studies in adult rodents have reported that systemic testosterone injections induced CPP in male rats and mice (de Beun et al., 1992; Alexander et al., 1994; Arnedo et al., 2000, 2002; Frye et al., 2001). In another animal model, it has been demonstrated that 15 days of administration of an AAS cocktail consisting of testosterone cypionate, nandrolone decanoate, and boldenone undecylenate, increased the rate of self-administration and enhanced the sensitivity to amphetamine challenge (Clark et al., 1996). However, in the same study, a 2 week treatment with MT had no effect on reward or performance of intracranial self-stimulation. In this light, Ballard and Wood (2005) have reported that in animals drostanolone and nandrolone tend to be self-administered (Ballard and Wood, 2005) and can cause CPP (Frye et al., 2002). Moreover, such effects can be prevented by dopaminergic antagonists (Schroeder and Packard, 2000) indicating that dopaminergic pathways are necessary for these behavioral outcomes. Indeed, the mesocorticolimbic circuitry, such as nucleus accumbens (NAc) and ventral tegmental area (VTA) are crucial for the reward system.

Parrilla-Carrero et al. (2009) investigated the rewarding effects of three different types of synthetic androgens differing in chemical structure and metabolism by using the CPP test in adult mice. They found that systemic injection of testosterone propionate and nandrolone decanoate, but not 17α-methyltestosterone, produced a dose-dependent shift in CPP suggesting that the rewarding properties of AAS might depend on their interaction with different pathways (Parrilla-Carrero et al., 2009). Very recently, the same research group has demonstrated nandrolone's failure to reward in adolescent mice (Martinez-Rivera et al., 2015). Although the literature reports that the adolescent brain is more sensitive to the reinforcing effects of drugs of abuse, this study suggests that such sensitivity may be drug dependent (Ernst et al., 2009; Galvan, 2010; Martinez-Rivera et al., 2015).

Packard et al. (1997) reported that testosterone induced CPP when directly injected into NAc (Packard et al., 1997). Similarly, Frye et al. (2002) showed that direct implants of testosterone or its metabolites (dihydrotestosterone, 3α-androstanediol) in the NAc shell induced a preference for the androgen-associated compartment, while no effect was observed with androgenic stimulation of the NAc core, suggesting a sub-region-specific functional role in reinforcement and reward pathway.

A growing body of evidence has shown the reinforcing effects of AAS using the experimental self-administration (oral, intravenous iv, intracerebroventricular icv) paradigm, which is considered as a model of addiction with the greatest face validity (Johnson and Wood, 2001; Wood, 2004; Frye, 2007; Frye et al., 2007). Wood (2002) demonstrated that gonadally intact adult male hamsters preferentially self-administer testosterone orally by using a food-induced drinking model (Wood, 2002). Although oral self-administration resembles oral AAS intake in humans, potential effects of taste solution or gut fill might present an inherent limitation on AAS oral consumption. Thus, Wood et al. (2004) used an operant chamber to train animals with chronic jugular cannulae and demonstrated an increase in testosterone iv self-administration compared to controls. Moreover, Syrian hamsters voluntarily consume testosterone through icv self-administration, suggesting that testosterone-reinforcing effects are centrally mediated (DiMeo and Wood, 2004; Wood, 2004). Ballard and Wood (2005) extended their research study on androgens and compared icv self-administration of four commonly abused AAS (nandrolone, drostanolone, oxymetholone, stanozolol) that differ in their method of administration, duration of action and metabolism. Results from this study showed that male hamsters preferentially self-administered nandrolone or drostanolone, which are two of the mostly used injectable androgens in humans. Conversely, animals failed to self-administer the orally active androgens oxymetholone or stanozolol, suggesting that injectable androgens may be more reinforcing than orally active steroids (Ballard and Wood, 2005).

To better understand the behavioral outcomes described above, various neurochemical studies have examined AAS effects on the monoaminergic system by measuring neurotransmitter and metabolite levels or by detecting receptors and enzyme alterations in key brain areas linked to the reward pathway. It has been reported that CPP induced by testosterone was blocked when adult male rats were directly injected into NAc with a D_1_-like or D_2_-like dopamine receptor antagonist (SCH23390 or sulpiride, respectively) (Schroeder and Packard, 2000). Sub-chronic administration of high AAS doses reduced dopamine D_1_-like receptor protein and mRNA levels in the NAc core and shell and increased D_4_-receptor mRNA expression in NAc, while D_2_-like receptors were up-regulated in the NAc core but down-regulated in the shell (Kindlundh et al., 2001b, 2003; Birgner et al., 2008a; Martinez-Rivera et al., 2015). An up-regulation of the dopamine transporter (DAT) protein was observed *in vivo* by a binding study using positron emission tomography (PET), in the striatum of male rat brain after chronic treatment with nandrolone (Kindlundh et al., 2002). Interestingly, Martinez-Rivera et al. (2015), observed no difference of D_1_-receptor protein expression in adolescent mice suggesting that the mesolimbic dopaminergic system during adolescence is immature or not sensitive to the rewarding response induced by nandrolone. Studies in Syrian hamsters suggested that testosterone reduced dopamine (DA) release in NAc (Triemstra et al., 2008). Likewise, our research group showed a reduction in DA content in NAc of rats treated for 4 weeks with nandrolone, changes which were accompanied by reduced hedonic-related behavior (Zotti et al., 2014). Furthermore, Birgner et al. (2007), in a microdialysis study, demonstrated that sub-chronic nandrolone decreased extracellular levels of DA metabolites (DOPAC and HVA) in rat NAc shell without affecting the release of DA. In line with these results, nandrolone was shown to reduce type A and B activity of monoamine oxidase (MAO) (Birgner et al., 2008b), although a previous study reported no effects of the drug on these enzymes activity in rats (Thiblin et al., 1999a). Further confirming the role of dopaminergic system in AAS effects on reward pathway, subchronic nandrolone has been shown to significantly down-regulate D_1_ receptors in the NAc and caudate putamen of rats, and to up-regulate D_2_-like receptors in the NAc core and VTA (Kindlundh et al., 2001b). In this regard, D_1_ and D_2_ receptors have been implicated in the reinforcing effects of drugs, as D_1_ is necessary for the acquisition of the effect and D_2_ crucial in mediating positive reinforcement (Missale et al., 1998). On the other hand, we have previously reported that stanozolol had no effect on DA content in NAc (Tucci et al., 2012). Findings regarding the impact of different AAS on brain reward function are summarized in Table 1.

**Table 1:** **Preclinical overview of the impact of different AAS on reward system**.

**Drug**	**Route and dose of administration**	**Impact on reward system**	**Species**	**References**
Testosterone	0.8–1.2 mg/kg s.c	↑ CPP	Mice	Arnedo et al., 2000
	1–2 mg/kg s.c			Arnedo et al., 2002
	0.75 mg/kg; 7.5 mg/kg i.p			Parrilla-Carrero et al., 2009
	0.5–1 mg/kg s.c	↑ self-administration	Rats	de Beun et al., 1992
	0.8–1.2 mg/kg s.c			Alexander et al., 1994
	1 μg icv infusion,	↑ self-administration	Hamsters	Wood et al., 2004
	50 μg iv,			DiMeo and Wood, 2004
	1–4 mg/ml oral self-administration			Wood, 2002
	2 μg icv infusion	↓ DA (NAc)	Hamsters	Triemstra et al., 2008
Nandrolone	0.75 mg/kg;	↑ CPP	Adult Mice	Parrilla-Carrero et al., 2009
	7.5 mg/kg i.p	↓ D1R (NAc)		Martinez-Rivera et al., 2015
	7.5 mg/kg i.p	No effect on CPP, no difference in D1R	Adolescent Mice	Martinez-Rivera et al., 2015
	1 μg/μl; 2 μg/μl icv self-administration	↑ self-administration	Hamsters	Ballard and Wood, 2005
	15 mg/kg i.m	↑ DAT	Rats	Kindlundh et al., 2002
		↓ D1R; ↓ D2R (NAc shell)		Kindlundh et al., 2001b, 2003
		↓ DOPAC, ↓ HVA		Birgner et al., 2007
		↓ MAO-A, ↓ MAO-B		Birgner et al., 2008b
	15 mg/kg s.c	↓ DA (NAc)	Rats	Zotti et al., 2014
	3 mg/kg; 15 mg/kg i.m	↑ D4R mRNA (NAc)	Rats	Birgner et al., 2008a
Stanozolol	1 μg/μl; 2 μg/μl icv self-administration	No effect on self-administration	Hamsters	Ballard and Wood, 2005
	15 mg/kg s.c	No effect on DA	Rats	Tucci et al., 2012
Methandrostenolone	1 mg s.c	No effect on intracranial self-stimulation	Rats	Clark et al., 1996
17α-methyltestosterone	0.75 mg/kg; 7.5 mg/kg i.p	No effect on CPP	Mice	Parrilla-Carrero et al., 2009

Contradictory neurochemical results have been reported regarding AAS effects on the serotonergic system. In particular, intranasal administration of testosterone has been shown to increase dopaminergic and serotonergic systems in rat neostriatum and NAc (de Souza Silva et al., 2009). Accordingly, nandrolone decanoate and oxymethenolone treatment enhanced 5-HT and 5-HIAA concentrations in rat cerebral cortex and hypothalamus, while decreased levels of 5-HT and 5-HIAA were observed in the striatum of nandrolone-treated rats (Thiblin et al., 1999a; Lindqvist et al., 2002; Tamaki et al., 2003). Moreover, it has been shown that AAS affects 5-HT receptor expression. In particular, sub-chronic nandrolone administration down-regulates 5-HT_1B_ and up-regulates 5-HT_2_ receptor density in rat brain (Kindlundh et al., 2003). In addition, McQueen et al. (1999) have demonstrated that serotonin transporter (SERT) mRNA-expressing cells in the dorsal raphe nucleus, as well as the density of SERT sites increase after sub-chronic treatment with testosterone (McQueen et al., 1999).

On the other hand, several studies have associated the endogenous opioid system to behaviors linked to reward and reinforcement (Gianoulakis, 2009). Thus, a number of experimental investigations have been carried out to ascertain whether AAS treatment modifies the levels of opioid peptides and their receptors in brain areas mediating reward. In particular, β-endorphin levels have been reported to significantly increase in the paraventricular thalamic nucleus and VTA of rats treated with AAS cocktails or nandrolone decanoate, respectively (Johansson et al., 1997; Harlan et al., 2000). In line with previous reports, chronic exposure to nandrolone decanoate has been linked to enhanced μ-, δ-, and κ-receptor binding in the hypothalamus, striatum, and midbrain periaqueductal gray (Johansson et al., 2000a). However, in the NAc shell and central amygdala of rats treated with the higher dose of nandrolone regimen, a down-regulation of κ-receptor binding, as measured by autoradiography has been demonstrated (Magnusson et al., 2009). Moreover, an increase in dynorphin converting enzyme-like activity was found only in the NAc of rats exposed to chronic nandrolone, suggesting an increased biosynthesis of dynorphin peptides, which, in turn, might affect basal DA levels in the NAc (Spanagel et al., 1992; Steiner and Gerfen, 1998; Magnusson et al., 2007).

## Other AAS pathways and reward

It is worth noting that AAS effects are commonly described after chronic or sub-chronic drug exposure. Indeed, acute subcutaneous testosterone administration failed to influence accumbal DA release (Triemstra et al., 2008). In this regard, it has been proposed that AAS effects on the reinforcement system may be DA-independent, as happens with other abuse substances such as ethanol and benzodiazepines. Moreover, AAS effects on mesolimbic dopamine might be indirect or rely on non-classic androgen-sensitive pathways. Thus, based on accumulated evidence, AAS have an addictive potential, especially in susceptible subjects.

As reported, many discrepancies need to be better clarified. First, it is important to clarify whether classic nuclear receptors are involved in these effects or if other mechanisms are also involved. Moreover, scientific evidence exists for fast actions of steroids acting on calcium channels, membrane receptors, second messengers and membrane fluidity (for a review see Foradori et al., 2008). In this regard, a recent *in vitro* study has shown that testosterone, by acting on membrane receptors, was able to increase hippocampal plasticity within 2 h, leading to increased spine density (Li et al., 2015). Sato et al. (2010) investigated the possible involvement of these types of receptors on reinforcement effect of AAS. In particular, their experiments demonstrated that animals, intact or carrying the testicular feminization mutation, preferentially self-administer dihydrotestosterone (DHT) and DHT conjugated to bovine serum albumin, DHT-BSA, which acts only on cell surface. These observations prompted the authors to conclude that androgen self-administration may be mediated by plasma membrane receptors (Sato et al., 2010). Accordingly, it has been postulated that classical genomic action of androgen may be not fast enough to assure reinforcement. In agreement to such hypothesis, the distribution of androgen receptors in NAc and VTA is resulted quite sparse (Kritzer and Creutz, 2008; Sato et al., 2008).

Nonetheless, it is worth to note that further signaling systems, other than dopaminergic or opioidergic, can be implicated in reward. Indeed, chronic nandrolone decanoate was found to down-regulate the NR1 subunit of NMDA receptors in NAc of treated rats (Le Greves et al., 1997). This finding led the Authors to hypothesize that AAS may thus sensitize reward mechanisms.

However, the number of studies investigating the effects of AAS on glutamatergic system in reward is still limited; hence, future investigations should be focused accordingly to clarify whether AAS reinforcement relies on non-classic pathways or on other signaling systems.

## AAS effects on other drugs of abuse

Clinical and epidemiological data have reported that the abuse of AAS in humans is often associated with the abuse of psychotropic drugs, such as cocaine, opiates, alcohol, cannabis, amphetamine, and 3,4-methylenedioxy-methamphetamine (MDMA). These surveys have suggested a role of AAS as a gateway to other dependency-inducing drugs (DuRant et al., 1995; Arvary and Pope, 2000; Kindlundh et al., 2001a; Kanayama et al., 2003a; Thevis et al., 2008). Based on these findings, different animal paradigms have been used to investigate AAS pre-exposure effects on neurochemical and behavioral response to other addictive substances. Consistent with reported higher alcohol intake in AAS abusers, increased voluntary alcohol consumption after cessation of AAS administration has also been observed in male adult rats (Johansson et al., 2000b). In line with these findings, corticotropin releasing factor modulation of GABAergic transmission in the amygdala seems to play a pivotal role in ethanol effects, suggesting that AAS might alter the sensitivity of these circuits and predispose to alcohol abuse (Roberto et al., 2004, 2010; Oberlander and Henderson, 2012). Chronic nandrolone decanoate administration has been found to significantly impair CPP induced by Δ9-tetrahydrocannabinol (THC) without affecting CB1 receptor binding. Interestingly, nandrolone administration increased THC abstinence precipitated by the CB1 cannabinoid antagonist rimonabant (Celerier et al., 2006).

Administration of supra-pharmacological doses of nandrolone decanoate has been shown to decrease the hyper-locomotion and stereotyped behavior induced by amphetamine and MDMA, in a dose-dependent manner (Kurling et al., 2008). Such behavioral outcomes have been corroborated by microdialysis results. In particular, nandrolone decanoate attenuated the effect of amphetamine and MDMA on DA baseline and DA metabolites levels in the NAc. However, the higher dose of nandrolone decanoate has enhanced the acute effects of MDMA-induced release of 5-HT, followed by exhaustion of neuronal 5-HT stores. Thus, high-dose nandrolone decanoate treatment might enhance neuron vulnerability to MDMA, leading to effects resembling MDMA neurotoxicity (Kurling et al., 2008). In addition, it has been demonstrated that the effects of amphetamine on the hippocampal and hypothalamic DOPAC/DA ratio were prevented by nandrolone decanoate, with no changes to DA baseline levels (Birgner et al., 2007). Likewise, it has been shown that pretreatment with nandrolone decanoate attenuates accumbal DA and 5-HT outflow, as well as the consequent stereotyped behavior induced by cocaine (Kurling-Kailanto et al., 2010; Kailanto et al., 2011). Nandrolone might decrease neurochemical and behavioral effects induced by cocaine via up-regulation of DAT and SERT binding sites. In these studies, the authors showed that changes in DA and 5-HT systems endure, even after a long recovery period from the last dose of nandrolone. This confirms the hypothesis that drug abuse causes long lasting changes in brain dopaminergic and serotonergic pathways (Kurling et al., 2008; Kailanto et al., 2011). These data are in line with earlier findings demonstrating that chronic cocaine and methamphetamine decreased D_2_-receptor and DAT expression during withdrawal and lasted up to 11 months after the last drug administration (Volkow et al., 1990, 2001a,b). Collectively, these results demonstrate that pre-treatment with nandrolone decanoate dose-dependently attenuates neurochemical and behavioral effects relating to the reward system induced by psychostimulant drugs. These findings indicate that such reduced dopaminergic and serotonergic activity in brain regions strictly involved in the reward system might represent the neurochemical substrate that could underlie a higher prevalence of illicit drug use among AAS abusers. Indeed, to achieve the desired effect of psychostimulant drugs, AAS users may require increased doses of these substances.

On the other hand, testosterone has been hypothesized to act as a partial agonist on the opiod system considering that, depending on type of receptors involved, steroid effects are brain region specific (Wood, 2008). As the reinforcing effects of opioids are thought to be mediated principally by μ- and δ-receptors (Peters and Wood, 2005), many data in the literature are available with regard to altered opioid receptor binding after AAS. In particular, nandrolone has been reported to increase binding of μ-, δ-, and κ-receptors in the hypothalamus, striatum, and midbrain periaqueductal gray (Johansson et al., 2000a), while reduced κ-receptors have been found in NAc (Johansson et al., 2000b). In addition, increased β-endorphin levels in the VTA (Johansson et al., 1997) and paraventricular thalamus (PVT) (Harlan et al., 2000) along with and higher β-endorphin fiber staining in bed nucleus of the stria terminalis and PVT (Menard et al., 1995) have been described. However, the total β-endorphin immunoreactivity is lower in arcuate nucleus (Menard et al., 1995).

On the other hand, nandrolone has been found to enhance morphine-induced hypothermia while testosterone increases the antinociceptive effect of a κ-agonist. However, contrasting data exist since no effects of AAS on morphine antinociception have been reported in other animal models (Negus et al., 2001; Celerier et al., 2003; Philipova et al., 2003). In fact, nandrolone pre-exposure has been shown to inhibit tolerance to antinociceptive properties of morphine and CPP induced by morphine in mice (Celerier et al., 2003) and rats (Philipova et al., 2003). Accordingly, pre-exposure to AAS has been shown to prevent morphine-induced striatal Fos expression (Harlan et al., 2000). High variability is present in findings linking AAS to opiate withdrawal. In monkeys no effect of AAS has been described for naloxone-precipitated morphine withdrawal paradigm, although Celerier et al. (2003) found that nandrolone increased withdrawal symptoms induced by naloxone in morphine-dependent mice. Moreover, the dysphoric effect mediated by nandrolone pre-treatment has been correlated to elevated striatal levels of dynorphin B, which in turn may account for the inhibition of dopaminergic activity in this brain region (Steiner and Gerfen, 1998; Johansson et al., 2000a). Finally, testosterone has been shown not to increase motivation for morphine (Cooper and Wood, 2014). Such discrepancies may rely on different AAS and schedule of treatment used, as well as different species or strain of animal used.

Although literature has been populated by many clinical or preclinical reports, many knots in the unraveling of deleterious addictive effects of AAS still need to be untied. Furthermore, taking into account that the use of these substances is becoming popular, especially among adolescents, a deeper knowledge of CNS effects of AAS is nowadays mandatory.

## Funding

This review was supported by PRIN 2011 (PT) from MIUR.

### Conflict of interest statement

The authors declare that the research was conducted in the absence of any commercial or financial relationships that could be construed as a potential conflict of interest.
